# Growth and body composition in children who are picky eaters: a longitudinal view

**DOI:** 10.1038/s41430-018-0250-7

**Published:** 2018-07-11

**Authors:** Caroline M. Taylor, Colin D. Steer, Nicholas P. Hays, Pauline M. Emmett

**Affiliations:** 1 Centre for Child and Adolescent Health, Bristol Medical School, University of Bristol, Bristol, UK; 2 Bristol Medical School, University of Bristol, Bristol, UK; 3 Nestlé Nutrition, La Tour-de-Peilz, Switzerland

**Keywords:** Nutrition, Epidemiology

## Abstract

**Background/objectives:**

Picky eating may be associated with higher risk of being underweight and poor growth over time or conversely, being overweight. Our aim was to investigate if children identified as picky eaters showed differences in height, weight and body composition from their non-picky peers.

**Subjects/methods:**

Picky eaters were identified in the Avon Longitudinal Study of Parents and Children cohort at 3 years of age. Height and weight were measured on seven occasions (age 7–17 years). Body composition was measured on five occasions by dual-energy x-ray absorptiometry (age 9–17 years). Participants were classified as *thin/normal/overweight or obese* at each age point using body mass index (BMI) classifications. Data were analysed with adjusted multiple regression analysis and mixed-design repeated measures ANOVA.

**Results:**

There was a main effect of being a picky child on height and weight (and on BMI and lean mass index (LMI) in boys) (lower in the picky children, all *p* ≤ 0.044), but not on percentage body fat or fat mass index (and not on BMI and LMI in girls) (all *p* > 0.2). The mean heights, weights and BMIs of picky eaters were consistently above the 50th centiles of reference growth charts. More than two-thirds of picky eaters were not thin at any age point. However, being a picky eater was predictive of being thin at a few age points.

**Conclusions:**

The growth trajectories of children who were picky eaters were reassuring. The prevalence of thinness amongst some picky eaters is notable, suggesting that some children may need specific early identification, intervention and growth surveillance.

## Introduction

Picky eating is generally defined as including an unwillingness to try new foods (food neophobia) together with strong food preferences and avoidance of some familiar foods [1, 2]. It can be identified with a subjective parental-completed questionnaire on facets of eating behaviour; estimates of prevalence range widely from 6 to 60% [2]. The prevalence seems to peak at about 3 years of age [3, 4].

Picky eating can lead to a higher risk of being underweight and having poor growth [5–11], or conversely of being overweight [12]. This may be driven by poor dietary variety in childhood [4, 13, 14], with rejection of vegetables being a common finding [15–18]. Intakes of vitamins and minerals, particularly those that are critical for growth such as iron and zinc, can be compromised [18, 19], although findings on energy intakes have been less consistent [16, 18–20]. However, as discussed by Berger et al. [21], the interpretation of most studies on growth in picky children is limited by their cross-sectional design, and so the possiblity of reverse casuality cannot be eliminated. There are very few longitudinal studies on measures of growth in children who are picky eaters: in studies limited to measures of prepubescent growth, picky children identified whilst they were preschoolers were more likely to be underweight and less likely to be overweight 2–4 years later [22, 23]. The longer term effects of picky eating on growth and body composition in pubescent or postpubescent adolescents have received even less attention: Berger et al. [21] found that girls who were persistent picky children studied from age 5 to 15 years of age were within the normal weight range and were less likely to be overweight than non-picky children, and not more likely to be underweight. In the context of child populations where overweight and obesity are common, it is possible that selective and limited eating by picky children provides some protection against these conditions, but at the expense of some aspects of dietary quality, particularly fruit and vegetable intake.

We have previously used data from the Avon Longitudinal Study of Parents in Children (ALSPAC), a longitudinal birth cohort study, to identify and characterise preschool picky children [2] and to describe diet and health outcomes [18, 24]. The aim of the present study was to investigate if children identified as picky eaters at 3 years of age in ALSPAC showed longitudinal differences in height, weight, body mass index (BMI) and body composition at ages between 7 and 17 years from their non-picky peers. The results will inform the need for early preventative intervention strategies for picky children and their caregivers.

## Methods

### The ALSPAC cohort

ALSPAC is a longitudinal population-based study investigating environmental and genetic influences on health, behaviour and development of children. All pregnant women in the former Avon Health Authority with an expected delivery date between April 1991 and December 1992 were eligible for the study; a total of 14,541 pregnant women were initially enrolled, resulting in a cohort of 14,062 live births with 13,988 alive at 1 year of age [25, 26]. Details of the informed consent process are described in Boyd, Golding et al. [25]. Further details of ALSPAC are available at www.bris.ac.uk/alspac, and the study website contains details of all the data that are available through a fully searchable data dictionary (http://www.bris.ac.uk/alspac/researchers/data-access/data-dictionary). Ethics approval for the study was obtained from the ALSPAC Ethics and Law Committee and the Local Research Ethics Committees.

### Defining picky eating in the ALSPAC cohort

The primary caregiver (usually the mother) received a series of postal self-completion questionnaires. The questionnaires are available from the study website (http://www.bristol.ac.uk/alspac/researchers/questionnaires/). A single question on picky eating was asked at 38 months. The question was: ‘Does your child have definite likes and dislikes as far as food is concerned?’ with possible responses No/Yes, quite choosy/Yes, very choosy. The responses for singleton cases were scored 0, 1 or 2 to describe the children as *not picky*, *somewhat picky* or *very picky*.

### Measurement of anthropometry

Growth data were collected by standardised routine measurements in annual clinics from ages 7 to 17 years (except ages 14 and 16 years). Age in months at clinic attendance was recorded.

Standing height was measured to the last complete millimetre using the Harpenden Stadiometer (Holtain Ltd, Crymych, UK) and weight was measured to the nearest 0.1 kg using the Tanita Body Fat Analyser (Model TBF 305, Tanita, Tokyo, Japan). Total body fat mass and total body lean mass was measured with dual-energy x-ray absorptiometry (Lunar Prodigy DXA scanner, GE Medical Systems, Madison, WI, USA) at ages 9, 11, 13, 15 and 17 years. Scans with anomalies (movement artefacts, artefacts caused by jewellery) were excluded. Within the three groups of picky children (*not picky*, *somewhat picky*, *very picky*) at each age, the children were categorised into BMI groups (thin (underweight)/normal/overweight/obese) using age- and sex-specific cut-offs [27, 28]. The three thinness (underweight) categories (grades 1–3) were elided to form a single category for thinness; the overweight and obese categories were also elided to form a single category.

### Additional data and confounders

A number of variables from the data collected from parental questionnaires or clinic visits were considered as potential confounders based on those used in previous studies in the literature and those with *p* < 0.01 in univariate analysis. These were: (1) maternal variables (maternal education, pre-pregnancy body mass index, maternal age, parity); (2) child variables (birthweight, being breastfed at 6 months, baseline BMI at 38 months, age at each clinic visit).

### Statistics

Data were analysed with SPSS version 23. There was no evidence of differential attrition in the three groups (*very picky*, *somewhat picky*, *not picky*) for participants who had complete data on anthropometry, body composition and confounders (Supplementary Table 1). Two datasets were prepared: (1) complete cases (picky eating variable at 38 months, complete set of anthropometry and body composition data, complete set of confounders); (2) multiple imputed data set (picky eating variable at 38 months plus at least one height or weight measurement) with 20 imputed datasets each (Multiple Imputation function in SPSS). Multiple imputation was evaluated because: (1) a complete case analysis is likely to have some degree of bias; (2) complete cases comprised about 25% of all cases having data on picky eating at 38 months, reducing the power of the models. However, the amount of missing data for height and weight was close to 50% at age 17 years, making the results from a dataset with multiple imputation potentially less reliable (Supplementary Table 2). Thus, results for multiple imputation are presented in the supplementary tables, with those for complete cases shown in the main tables.

For anthropometric and body composition variables at each age point, ANOVA was used to compare *very picky* (score 2) with *not picky* (score 0) by sex for all cases. Group mean values for height, weight and BMI were plotted on UK growth centile charts [29]. Adjusted mixed-design repeated measures ANOVA in SPSS (GLM procedure) was used to investigate the effect of picky eating on anthropometry and body composition with time for boys and girls separately. Linear regression analysis was used to model the differences in anthropometric variables between *very picky* and *not picky* children, with adjustment for confounders. Adjusted logistic regression analysis was used to model the odd of being thin (underweight; all grades) or being overweight/obese compared with having a normal BMI at each age.

### Code availability

Computer code is not available.

## Results

The study flow chart is shown in Supplementary Fig. 1. Demographic characteristics of the participants in the three categories of picky eating are shown in Taylor, Wernimont et al. [2].

**Fig. 1:**
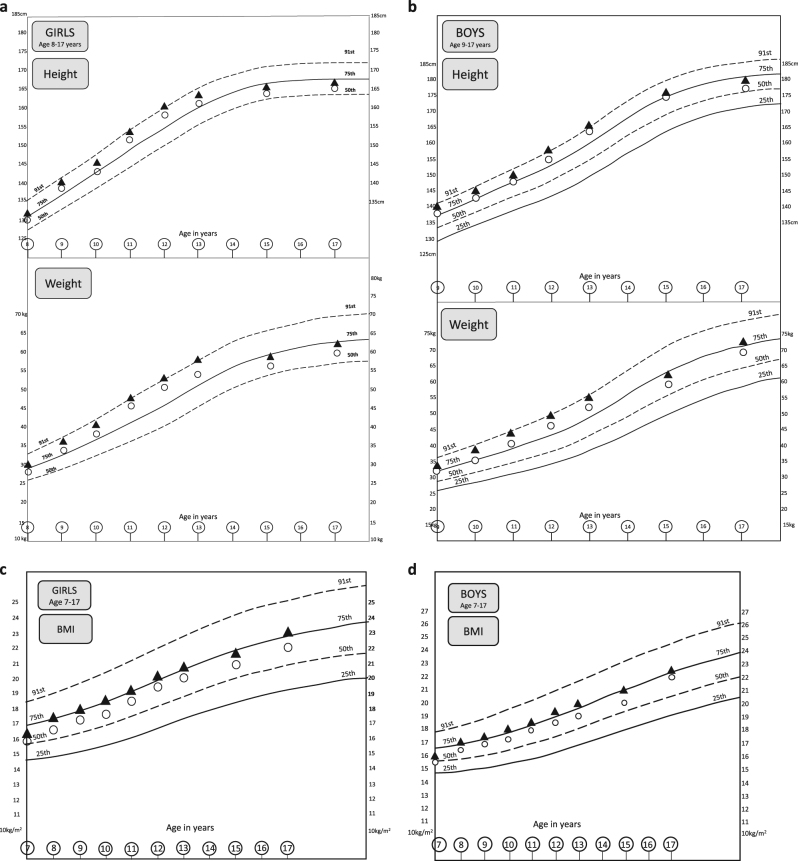
Centile trajectories for height and weight for girls and boys (**a**, **b**) and BMI for girls and boys (**c**, **d**) (complete cases) in ALSPAC. White circles, *very picky children* (boys *n* = 136, girls *n* = 157); black triangles, *not picky* children (boys *n* = 404, girls *n* = 468). *p* < 0.005 for all data pairs (ANOVA). Centile charts © Royal College of Paediatrics and Child Health 2013, reproduced with permission [29]

Height, weight and BMI for both boys and girls tended to track along centile lines when plotted on the growth charts; the trajectories of the *very picky* children were consistently about 5–10 centile points below those of the *not picky* children, but there were no age points in either group where the mean values were below the 50th centile (Fig. 1).

The percentage body fat was weakly significantly lower for boys and for girls who were *very picky* compared with *not picky* at three time points by about 1.5 percentage points (Table 1). The results were similar for fat mass index (FMI) for which the difference was about 0.5 kg/m^2^. For lean mass index (LMI), differences were also apparent for both boys and girls, being lower for *very picky* from age 11 by about 0.4 kg/m^2^ in boys and 0.2 kg/m^2^ in girls.

**Table 1:** Body composition from age 7 to 17 years for children identified as *very picky* vs. *not picky* children at 38 months in the ALSPAC cohort

Age (years)	Body fat (%)		FMI (kg/m^2^)		LMI (kg/m^2^)	
	Not picky	Very picky	Not picky	Very picky	Not picky	Very picky
Boys						
9	19.4 (18.5, 20.3)	17.7 (16.3, 19.2), *p* = 0.054	3.4 (3.1, 3.7)	2.95 (2.6, 3.3), *p* = 0.049	12.5 (12.2, 12.8)	12.1 (11.5, 12.6), *p* = 0.131
11	23.0 (22.1, 23.9)	21.7 (20.2, 23.5), *p* = 0.143	4.5 (4.3, 4.8)	2.95 (2.6, 3.3), *p* = 0.079	13.4 (13.3, 13.5)	13.0 (12.9, 13.2), *p* = 0.001
13	18.9 (18.0, 19.9)	17.3 (15.8, 18.7), *p* = 0.074	4.0 (3.7, 4.3)	3.48 (3.1, 3.9), *p* = 0.043	15.0 (14.9, 15.2)	14.7 (14.5, 14.9), *p* = 0.040
15	16.8 (16.0, 17.7)	15.2 (13.8, 16.7), *p* = 0.065	3.8 (3.5, 4.1)	3.25 (2.8, 3.7), *p* = 0.043	16.4 (16.2, 16.5)	15.9 (15.7, 16.2), *p* = 0.003
17	17.9 (17.0, 18.8)	17.5 (15.9, 19.3), *p* = 0.471	4.4 (4.0, 4.7)	4.07 (3.6, 4.6), *p* = 0.355	17.3 (17.1, 17.4)	16.9 (16.6, 17.1), *p* = 0.007
Girls						
9	25.5 (24.7, 26.4)	24.6 (23.2, 26.1), *p* = 0.305	4.6 (4.3, 4.8)	4.22 (3.8, 4.6), *p* = 0.188	11.6 (11.3, 11.9)	11.4 (10.9, 11.9), *p* = 0.565
11	27.8 (27.0, 28.5)	26.7 (25.4, 28.0), *p* = 0.158	5.5 (5.2, 5.7)	5.07 (4.7,5.4), p = 0.079	12.8 (12.7, 12.9)	12.5 (12.3, 12.7), *p* = 0.010
13	28.7 (28.0, 26.0)	27.2 (25.4, 28.3), *p* = 0.036	6.2 (5.9, 6.4)	5.62 (5.3, 6.0), p = 0.020	13.5 (13.4, 13.6)	13.3 (13.4, 13.6), *p* = 0.040
15	30.8 (30.1, 31.5)	29.3 (28.2, 30.5), *p* = 0.034	6.9 (6.7, 7.2)	6.36 (5.9, 6.8), *p* = 0.024	13.7 (13.3, 13.8)	13.5 (13.3, 13.6), *p* = 0.031
17	33.3 (32.5, 34.0)	31.9 (30.8, 33.1), *p* = 0.066	7.9 (7.6, 8.3)	7.26 (6.8, 7.7), *p* = 0.025	13.9 (13.8, 14.0)	13.7 (13.5, 13.9), *p* = 0.018

Data shown for complete cases

Data presented as mean (95% CI)

*Very picky*: boys *n* = 136, girls *n* = 157; *Not picky*: boys *n* = 401, girls *n* = 468

Data points for height, weight and BMI shown in Fig. 1

Mixed-design repeated measures ANOVA with adjustment for confounders showed an effect of being a *very picky* eater compared with *not picky* at 38 months on height and weight in boys and girls (Table 2). For LMI and BMI, there was an effect in boys, but not in girls. There was no effect on percentage body fat or FMI in boys or girls. Levene’s test for the assumption of homogeneity of variance was met in each case.

**Table 2:** Adjusted models of effect of being a *very picky* eater at age 38 months on anthropometric variables from age 7 to 17 years in the ALSPAC cohort (multiple linear regression analysis and repeated measures ANOVA): complete cases

Age (years)	Unstandardised B coefficient (95% CI)	*P* value	Unstandardised B coefficient (95% CI)	P value	Unstandardised B coefficient (95% CI)	P value
	Height (cm)		Weight (kg)		BMI (kg/m^2^)	
Boys (*n* = 537)						
7	−1.47 (−2.42, −0.52)	0.002	−0.99 (−1.68, −0.29)	0.006	−0.24 (−0.55, 0.06)	0.120
8	−1.63 (−2.67, −0.59)	0.002	−1.63 (−2.67, −0.60)	0.002	−0.28 (−0.65, 0.09)	0.132
9	−1.80 (−2.91, −0.70)	0.001	−1.55 (−2.71, −0.40)	0.008	−0.35 (0.79, 0.10)	0.125
10	−1.82 (−2.97, −0.67)	0.002	−2.10 (−3.43, −0.75)	0.002	−0.57 (−1.07, −0.07)	0.026
11	−1.87 (−3.14, −0.60)	0.004	−2.21 (−3.80, −0.62)	0.007	−0.52 (−1.06, 0.01)	0.058
12	−1.94 (−3.43, −0.44)	0.011	−2.48 (−4.29, −0.67)	0.007	−0.54 (−1.10, 0.24)	0.060
13	−1.77 (−3.36, −0.09)	0.035	−2.85 (−4.81, −0.88)	0.005	−0.65 (−1.20, −0.10)	0.020
15	−1.75 (−3.15, −0.35)	0.015	−3.47 (−5.54, −1.40)	0.001	−0.77 (−1.34, −0.20)	0.008
17	−1.56 (−2.75, −0.37)	0.010	−2.61 (−4.97, −0.24)	0.031	−0.47 (−1.14, 0.20)	0.172
R-M ANOVA		0.003		0.003		0.044
Girls (*n* = 625)						
7	−1.47 (−2.35, −0.59)	0.001	−0.93 (−1.62, −0.24)	0.008	−0.22 (−0.51, 0.08)	0.154
8	−1.78 (−2.73, −0.84)	0.001	−1.78 (−2.73, −0.84)	< 0.001	−0.19 (−0.54, 0.16)	0.289
9	−1.77 (−2.80, −0.73)	0.001	−1.20 (−2.33, −0.07)	0.038	−0.17 (−0.59, 0.26)	0.435
10	−1.86 (−2.99, −0.73)	0.001	−1.25 (−2.57, 0.06)	0.061	−0.13 (−0.59, 0.33)	0.573
11	−1.82 (−3.05, −0.59)	0.004	−1.32 (−2.86, 0.21)	0.091	−0.14 (−0.64, 0.37)	0.596
12	−1.55 (−2.70, −0.40)	0.009	−1.41 (−3.04, 0.22)	0.090	−0.21 (−0.73, 0.31)	0.436
13	−1.37 (−2.44, −0.30)	0.012	−1.48 (−3.08, 0.12)	0.070	−0.22 (−0.74, 0.30)	0.404
15	-1.09 (−2.13, −0.04)	0.041	−1.55 (−3.21, 0.12)	0.069	−0.29 (−0.84, 0.25)	0.291
17	−0.89 (−1.95, 0.18)	0.103	−1.49 (−3.38, 0.40)	0.122	−0.30 (−0.9, 0.32)	0.341
R-M ANOVA		0.002		0.043		0.376

	Body fat (%)		FMI (kg/m^2^)		LMI (kg/m^2^)	
Boys (*n* = 537)						
9	−1.25 (−2.90, 0.39)	0.135	−0.35 (−0.80, 0.10)	0.135	−0.39 (−1.00, 0.22)	0.206
11	−0.86 (−2.58, 0.86)	0.327	−0.29 (−0.75, 0.18)	0.232	−0.25 (−0.44, −0.06)	0.010
13	−0.92 (−2.71, 0.87)	0.312	−0.32 (-0.82, 0.18)	0.204	−0.30 (−0.59, −0.02)	0.036
15	−1.18 (−2.85, 0.51)	0.171	−0.38 (−0.87, 0.10)	0.126	−0.36 (−0.65, −0.07)	0.014
17	−0.21 (−1.94, 1.54)	0.817	−0.13 (−0.70, 0.44)	0.668	−0.34 (−0.64, −0.05)	0.024
R-M ANOVA		0.316		0.238		0.009
Girls (*n* = 625)						
9	0.27 (−1.28, 1.83)	0.305	0.01 (−0.27, 0.10)	0.374	−0.02 (−0.59, 0.56)	0.954
11	0.03 (−1.31, 1.38)	0.963	−0.02 (−0.42, 0.37)	0.909	−0.13 (0.32, 0.06)	0.177
13	−0.44 (−1.68, 0.81)	0.493	−0.16 (−0.27, 0.25)	0.451	−0.08 (−0.27, 0.11)	0.398
15	−0.44 (−1.68, 0.81)	0.493	−0.15 (−0.60, 0.29)	0.501	−0.12 (−0.31, 0.07)	0.214
17	−0.23 (−1.53, 1.08)	0.731	−0.16 (−0.68, 0.36)	0.549	−0.13 (−0.33, 0.07)	0.191
R-M ANOVA		0.780		0.621		0.360

Multiple regression analyses and R-M ANOVA adjusted for child age at each clinic visit, maternal age, maternal education, maternal BMI, birthweight, parity, breastfeeding, baseline BMI at age 38 months, age at BMI measurement

Reference: *not picky* eater at 38 months

R-M ANOVA, repeated measures ANOVA

In adjusted models of the association of anthropometric variables with very picky eating, there was evidence of strong negative associations with height in boys and girls. The models predicted that male and female *very picky* children were about 1.5–2.0 cm and 1.0–1.5 cm shorter, respectively, than *not picky* children at each age, although the difference tended to decrease and weaken in girls aged 12 years onwards. There were also strong negative associations for weight in boys, but the associations for girls were weak from the age of 10 years onwards (Table 2). The models predicted that male and female *very picky* children were about 1.5–2.5 kg and 1.0–1.5 kg lighter, respectively, than *not picky* children at each age. There was no evidence for any associations of percentage body fat or FMI with being *very picky*. Male *very picky* children had a lower LMI than *not picky* children at all age from 11 years onwards by about 0.1 kg/m^2^, but there was no evidence for any differences in girls.

The prevalence of thinness was greater in the *somewhat picky* and the *very picky* children than the *not picky* children at each age point (Table 3). The prevalence of overweight/obesity was conversely less in the *somewhat picky* and *very picky* children at most age points (Table 3). *Very picky* eating was associated with the odds of being thin at ages 7, 9, 10, 15 and 17 compared with normal weight in adjusted models (Table 3). There was no evidence that being a *somewhat picky* or *very picky* child increased the odds of being overweight/obese, except at the age 8 years for *very picky* children (Table 3). *Very picky* children tended to be thin at more age points than *not picky* children (Table 4), but nearly 70% of them were never thin at any age point.

**Table 3:** Prevalence of thinness and overweight/obesity with picky eating category at 38 months and odds of being thin or overweight/obese at 7–17 years in the ALSPAC cohort, complete cases

Age (years)	Prevalence, *n* (%)			Odds ratio (95% CI) (reference normal weight)		
	Not picky (*n* = 869)	Somewhat picky (*n* = 837)	Very picky (*n* = 293)	Not picky (ref)	Somewhat picky	Very picky
Thinness						
7	54 (6.2%)^a^	72 (8.7%)^ab^	34 (11.6%)^b^	–	1.19 (0.80, 1.76), *p* = 0.391	1.69 (1.05, 2.75), *p* = 0.032
8	27 (3.1%)^a^	52 (6.3%)^b^	18 (6.1%)^b^	–	1.91 (1.16, 3.14), *p* = 0.011	1.80 (0.95, 3.42), *p* = 0.070
9	48 (5.5%)^a^	76 (9.2%)^b^	31 (10.6%)^b^	–	1.50 (1.01, 2.23), *p* = 0.046	1.77 (1.07, 2.92), *p* = 0.027
10	47 (5.4%)^a^	77 (9.3%)^b^	34 (11.6%)^b^	–	1.58 (1.06, 2.65), *p* = 0.023	2.09 (1.28, 3.40), *p* = 0.003
11	56 (6.4%)^a^	80 (9.6%)^b^	31 (10.6%)^b^	–	1.39 (0.95, 2.02), *p* = 0.090	1.54 (0.95, 2.51), *p* = 0.081
12	65 (7.5%)^a^	88 (10.6%)^b^	35 (11.9%)^b^	–	1.29 (0.91, 1.83), *p* = 0.156	1.49 (0.95, 2.35), *p* = 0.086
13	16 (1.8%)^a^	31 (3.7%)^b^	10 (3.4%)^ab^	–	1.78 (0.96, 3.38), *p* = 0.068	1.59 (0.70, 3.64), *p* = 0.268
15	133 (15.3%)^a^	173 (20.8%)^b^	72 (24.6%)^b^	–	1.27 (0.97, 1.66), *p* = 0.081	1.56 (1.10, 2.22), *p* = 0.012
17	43 (4.9%)^a^	72 (8.7%)^b^	29 (9.9%)^b^	–	1.47 (0.98, 2.22), *p* = 0.066	1.87 (1.11, 3.14), *p* = 0.018
Normal					Ref	Ref
Overweight/obese						
7	127 (14.6%)^a^	98 (11.8%)^ab^	28 (9.6%)^b^	–	1.05 (0.57, 1.91), *p* = 0.887	0.74 (0.27, 2.05), *p* = 0.568
8	175 (20.1%)^a^	141 (17.0%)^ab^	41 (14.0%)^b^	–	1.02 (0.57, 1.81), *p* = 0.952	0.61 (1.28, 1.57), *p* < 0.001
9	171 (19.6%)^a^	130 (15.7%)^b^	43 (14.7%)^ab^	–	0.94 (0.51, 1.76), *p* = 0.857	0.93 (0.36, 2.40), *p* = 0.929
10	178 (20.4%)^a^	137 (16.5%)^b^	49 (16.7%)^ab^	–	0.93 (0.52, 1.65), *p* = 0.798	0.69 (0.25, 1.87), *p* = 0.463
11	173 (19.9%)^a^	146 (17.7%)^a^	50 (17.1%)^a^	–	0.90 (0.49, 1.66), *p* = 0.901	1.03 (0.42, 2.50), *p* = 0.952
12	183 (21.0%)^a^	137 (16.5%)^b^	49 (16.7%)^ab^	–	0.77 (0.42, 1.39), *p* = 0.380	0.67 (0.25, 1.81), *p* = 0.434
13	232 (26.7%)^a^	174 (21.0%^b^	53 (18.1%)^b^	–	0.89 (0.41, 1.95), *p* = 0.776	1.60 (0.76, 3.39), *p* = 0.220
15	115 (13.2%)^a^	85 (10.2%)^b^	27 (9.2%)^ab^	–	0.76 (0.37, 1.56), *p* = 0.458	0.61 (0.17, 2.15), *p* = 0.442
17	172 (19.7%)^a^	123 (14.8%)^b^	49 (16.7%)^ab^	–	0.71 (0.44, 1.15), *p* = 0.159	0.90 (0.44, 1.85), *p* = 0.770

Cole et al. 2000, 2007 BMI classifications [27, 28]: Cole Grade 1, 2 and 3 thinness elided; overweight and obesity elided

Multiple regression analyses adjusted for child age at each clinic visit, maternal age, maternal education, maternal BMI, birthweight, parity, breastfeeding, baseline BMI at age 38 months, actual age at baseline BMI measurement

^a,b^Values with superscript letters that are unlike in a row are statistically significantly different at *p* < 0.05

**Table 4:** BMI classification data at all time points from age 7 to 17 years by picky eating category at 38 months in the ALSPAC cohort, complete cases

	Not picky	Somewhat picky	Very picky
Thin at 0 time points	614 (80.0%)	552 (73.7%)	176 (69.6%)
Thin at 1 time points	68 (8.9%)	67 (8.9%)	19 (7.5%)
Thin at 2 time points	26 (3.4%)	41 (5.5%)	17 (6.7%)
Thin at ≥3 time points	60 (7.8%)	90 (11.9%)	41 (16.2%)

Values are n (%); Chi square *p* = 0.005; Cole et al. 2000, 2007 BMI classifications [27, 28]: Cole Grade 1, 2 and 3 thinness elided

For a comparison of results from the imputed dataset with complete case analyses, see Supplementary text.

## Discussion

In the group of *very picky* children identified at age 3 years in ALSPAC, we found evidence for differences in growth and body composition from age 7 to age 17 years in both boys and girls compared with *not picky* children. However, the mean heights, weights and BMIs of the *very picky* children were consistently above the 50th centiles of UK reference growth charts, which are based on the WHO Child Growth Standards, indicating that there is no great need for concern overall. There was no evidence of an increased likelihood of overweight or obesity in either the *very picky* or *somewhat picky* children. Nonetheless, almost one-fifth of the *very picky* children were thin at three or more age points compared with less than one-tenth of *not picky* children, although nearly three-quarters of *very picky* children were never thin at any age point.

There have been very few longitudinal studies on growth in picky children. However, in the few studies to date, there is emerging evidence of picky eating being predictive of thinness and/or protective against becoming overweight [22, 23]. The only study to our knowledge to include data from teenagers is that of Berger et al. in the USA: girls identified as persistent picky children assessed biannually had lower BMIs than non-picky children at every age point from 5 to 15 years of age and were less likely to become overweight in teenage years. However, picky children were within a normal weight range, tracking along the 50th centile for BMI, while the non-picky children tracked along the 65th centile [21]. We have been able to extend the work of Berger et al. by including boys as well as girls, extending the top of the age range from 15 to 17 years. Our findings are broadly in line with those of Berger et al. [21] in that the picky children in our study also tracked about 10–15 BMI centile points below that of the *not picky* children. However, our *picky* children tracked on about the 75th centile rather than the 50th centile. There are several possible reasons for this. (1) The position of the data on the centiles is higher than would be expected in the ALSPAC cohort [30] and may be related to selection bias for complete cases and/or selection bias in the caregivers who chose to answer the phenotyping question at 38 months. (2) Berger et al. [21] used the US CDC BMI chart, whereas we used the UK RCPCH chart: the latter is a growth reference chart describing how certain children grew at a specific place at a certain time, whereas the former describes the growth of healthy children under optimal conditions. Thus, the CDC charts tend to represent a more overweight population than the UK charts. Other differences include the method used to classify the children as being under- or overweight. Berger et al. [21] classified underweight as <3rd centile and overweight as ≥85th centile; we used the BMI cut-offs of Cole et al. [27, 28], who used data from a reference population from a heterogeneous mix of surveys from different countries to identify cut-offs in children. In addition the statistical methods in the two studies were somewhat different: the main advantage of the mixed modelling approach used by Berger et al. [21] is that it enables retention of cases with missing data. We addressed this by also including analyses of a dataset with multiple imputed data, but the proportion of missing data was possibly too high to allow confidence in the results. Finally, Berger et al. [21] did not include any potential confounders in their model, whereas we included an appropriate range of confounders.

This study is the first to our knowledge to include detailed longitudinal data on body composition in *very picky* children compared with *not picky* children. de Barse et al. [22] found a lower fat-free mass at 6 years in picky children than non-picky children. We found a suggestion of a lower LMI in *very picky* children in crude analyses in boys and girls, but there was only evidence for a difference in boys in adjusted regression models. As discussed by de Barse et al. [22], this is of potential concern in that high muscle mass and muscle strength are thought to have beneficial effects on metabolic and cardiovascular health. This requires further investigations with the inclusion of functional measures of muscle strength in picky children.

Deficits in nutrient intakes could underlie adverse effects on the child’s growth and development, with this outcome driven by a diet restricted in quality and/or quantity. Energy intake was not different between the two groups at 3 years of age in this cohort [18], consistent with the findings of Galloway et al. [19], but other studies have found that picky children consumed less [16] or more energy [20] than controls. Key micronutrients that are essential for growth have been shown to be low in the diets of picky eaters [31, 32], including in this cohort [18]. There is evidence for continued differences in diet at 10 and 13 years of age, particularly for meat, fruit and vegetables, between picky eaters and non-picky eaters in this cohort [33]. It is notable that of those children who were picky eaters at 3 years, the behaviour persisted in 47% at 4.5 years and in 40% at 5.4 years (using an identical question to identify picky eating at each age point) [2].

Our findings are in general reassuring for parents of most picky children. Most studies on picky eating have been carried out in developed countries, mainly the USA, where it is likely that the general population of children tend to be obese or overweight. If the overall child population tended to track along the 50th centiles for height, weight and BMI, rather than a higher centile as in the present study, then it is possible that being picky would be shown to have detrimental effect on growth. However, picky eating in this study was associated with an increased likelihood of being thin (underweight), and there was evidence that for a proportion of picky children, the thinness was persistent. The challenge may be to identify this subgroup of picky children early and to develop interventions to prevent thinness.

There are several strengths of the present study. (1) We used an unambiguous question about child choosiness that is similar to ones used in several recent studies [34–38], although it did not cover the full range of ‘picky eating’ traits as defined in some other studies [39–41]. A strength of this measure is that the question did not invite the parents to define picky eating for themselves. (2) Few databases include reliable longitudinal clinical measurements of height and weight, with a range of confounders. Even fewer include measures of body composition. (3) We were able to include both boys and girls, as the most comparable study, that of Berger et al. [21], did not include any boys. (4) There was no evidence for differential attrition between the three groups of picky children.

There are also a number of limitations. (1) Picky eating behaviour was identified at a single age point, and this does not capture whether it was a brief phase or sustained behaviour. (2) The results may not be generalisable to other populations and may apply only to a relatively overweight/obese population. (3) There may be selection bias in complete cases. (4) The number of complete cases was relatively small, and the number of cases of thinness (underweight) was particularly small. (5) There are likely to be confounders that were not able to account for. (6) We were not able to take differences in the timing of puberty into account (together with selection bias, this may partially explain the deviation of the growth trajectories of both picky eaters and non-picky eaters from their tracking centiles in late adolescence).

In conclusion, we found that in this group of picky children, mean weight, height and BMI trajectories did not indicate growth faltering compared with their non-picky peers on UK growth reference charts. The results could be of more concern in a population tending to be of normal weight or underweight, as the trajectories of the picky eaters might then fall below the 50th centiles. However, within the *very picky* children almost one-fifth were thin at three or more time-points between 7 and 17 years of age. Early identification of these children and the development of interventions remain a challenge.

## Electronic supplementary material

Supplementary material
