# Supplementary material for: Growth and body composition in children who are picky eaters: a longitudinal view
Source: Eur J Clin Nutr. 2018 Jul 11;73(6):869–78. doi: 10.1038/s41430-018-0250-7 (PMC6215483; doi:10.1038/s41430-018-0250-7)
Supplement: Supplementary file 1 — Supplementary material [file 41430_2018_250_MOESM1_ESM.docx]

**Supplementary Text**

**Comparison of complete case analyses with all cases and imputed dataset analyses**

Supplementary Figure 2 shows data from all cases (not categorised by picky eating score) plotted on the centile charts in comparison with the complete case data: as data from both databases track the centiles closely together, there is no evidence for bias in using complete cases rather than all cases.

Results using the imputed dataset are shown in Supplementary Tables 3 and 4: a disadvantage of the imputed dataset is that it does not provide 95% CI and p values from ANOVA, making direct comparisons with analyses of the complete cases difficult. However, some of the difference in values between the *not picky* and *very picky* groups were not as pronounced in the imputed dataset as in the complete cases dataset (Table 1 and Figure 1 vs Supplementary Table 3). Similarly, in adjusted regression models, the B coefficients tended to be greater using the complete cases dataset; the patterns of significance tended to be similar with the exception of BMI in girls, which was consistently non-significant in the complete cases and generally strongly significant in the imputed dataset. This was driven by stronger associations for weight in the imputed dataset (Table 2 and Supplementary Table 4).

**Supplementary Figure 1** Study flow chart

Pregnant women enrolled into ALSPAC

n=14,541

Height and weight measures at 7, 8, 9, 10, 11, 12, 13, 15, 17 years

Body composition measures (DXA) at 9, 11, 13, 15, 17 years

For sample numbers at each age point (all cases, complete cases and multiple imputation) and attrition rates see Supplementary Table 1

Score 0: Not a picky eater

n=4448 (45.2%)

Score 3: Very picky eater

n=1448 (14.7%)

Score 1: Somewhat picky

n=3848 (40.1%)

Excluded: multiple births

n = 375

Picky eating question answered
at 38 months

n=9844

Live births

n=14,062

Alive at 1 year

n=13,988

Eligible participants

n = 13,613

Excluded: multiple births

n = 375

Score 3: Picky eater

n=1448 (14.7%)

Height and weight measures at 7, 8, 9, 10, 11, 12, 13, 15, 17 years

Body composition measures (DXA) at 9, 11, 13, 15, 17 years

For sample numbers at each age point (all cases, complete cases and multiple imputation) and attrition rates see Supplementary Table 1

**
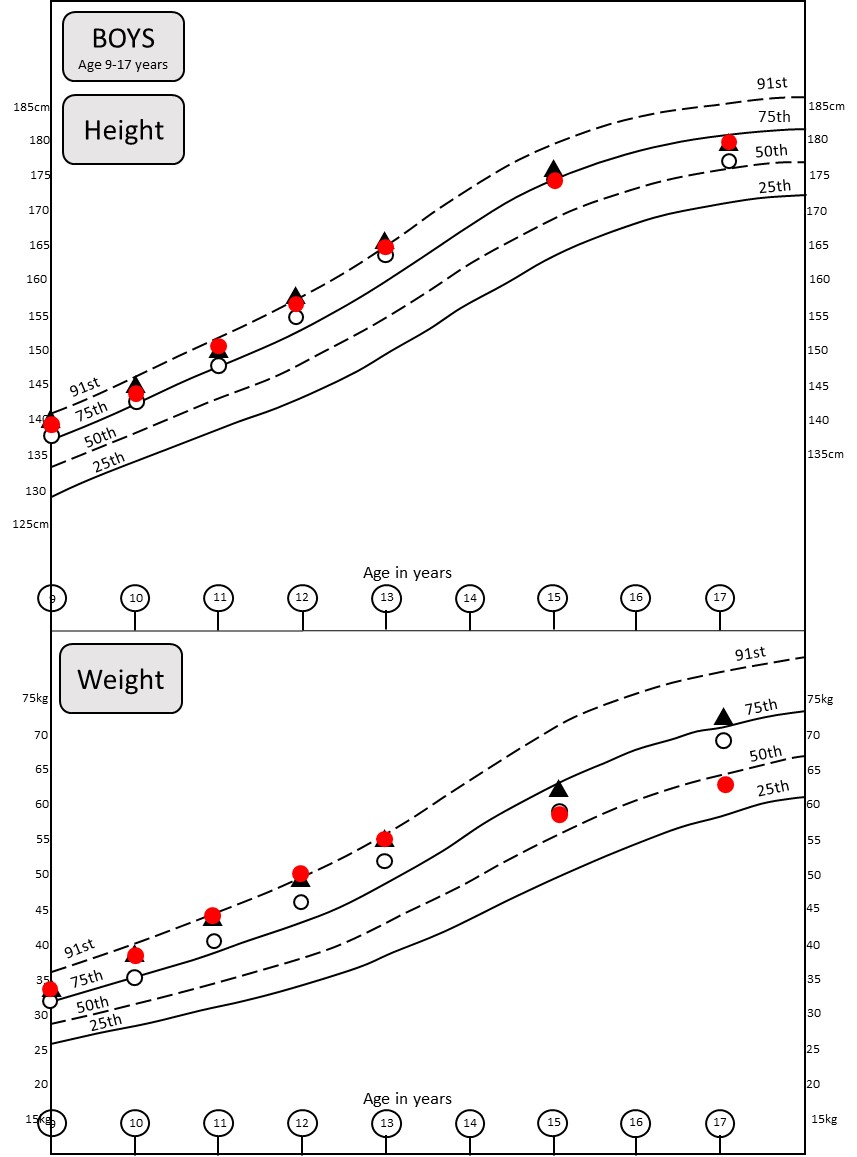

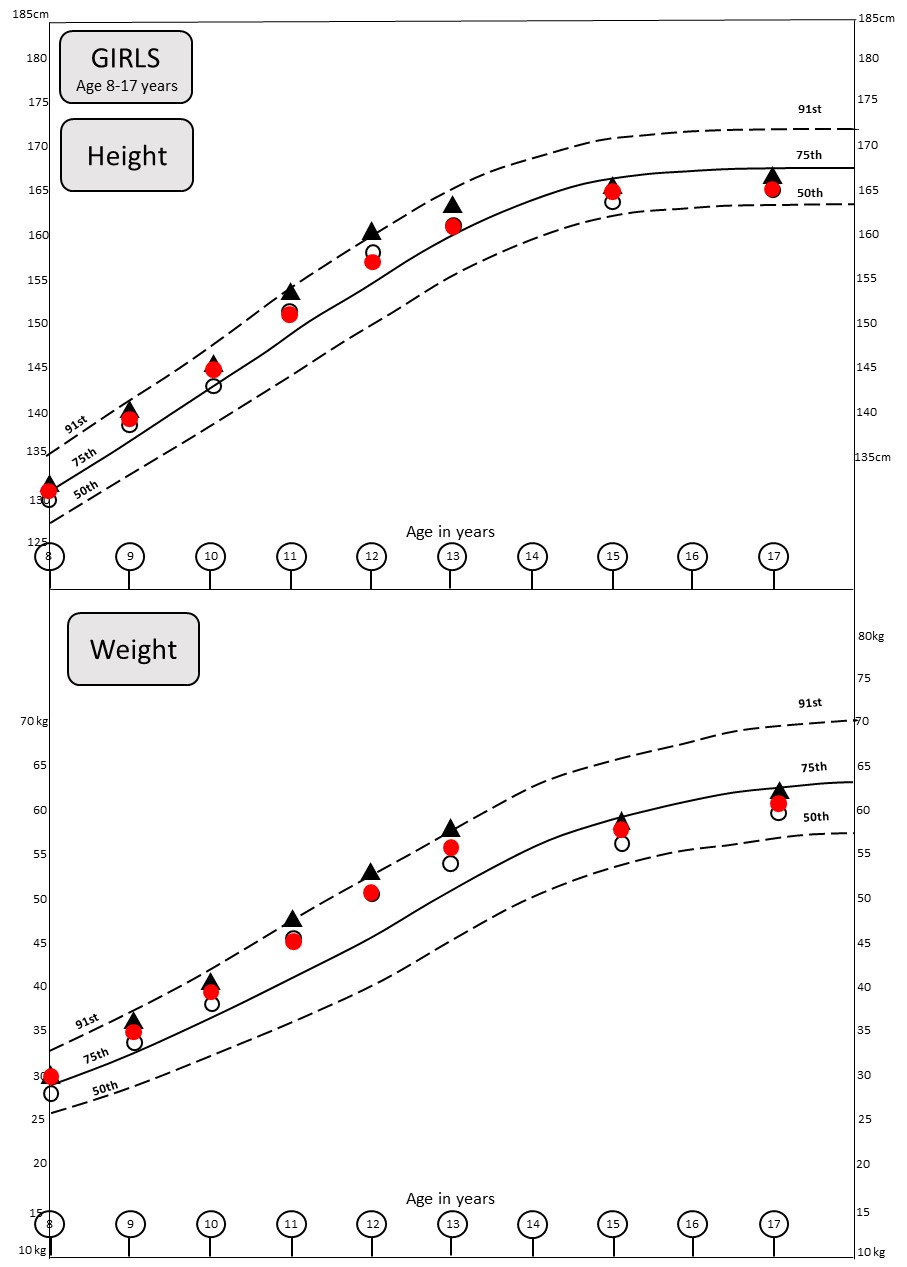
**

**Supplementary Figure 2**Centile trajectories for height and weight for girls and boys (a, b) and BMI for girls and boys (c, d) (complete cases: white circles and black triangle; all cases: red circles). White circles, *very picky* children, black triangles, *not picky* children. *Very picky* children: boys n=136, girls n=157; *not picky* children: boys n=404, girls n=468. All cases shown as combined *very picky/somewhat picky/not picky* groups. Centile charts © Royal College of Paediatrics and Child Health 2013, reproduced with permission.

**Supplementary Table 1** Sample numbers for children classified by picky eating score at age 38 months for anthropometric and body composition data from age 7 to 17 years in ALSPAC

| **Data** | **Picky eating score at 38 months** | | | **Chi square for difference from distribution of values at 38-month baseline (p value)** |
| --- | --- | --- | --- | --- |
|  | **0 (Not)** | **1 (Somewhat)** | **2 (Very)** |  |
| Picky eating score at 38 months | 4448 (100.0%) | 3848 (100.0%) | 1448 (100.0%) |  |
| Height/weight/BMI |  |  |  |  |
| 7 | 2994 (67.3%) | 2708 (70.4%) | 1031 (71.2%) | 0.316 |
| 8 | 2652 (59.6%) | 2466 (64.1%) | 902 (62.3%) | 0.125 |
| 9 | 2807 (63.1%) | 2564 (66.6%) | 948 (65.5%) | 0.246 |
| 10 | 2762 (62.1%) | 2536 (65.9%) | 920 (63.5%) | 0.292 |
| 11 | 2625 (59.0%) | 2385 (62.0%) | 913 (63.1%) | 0.253 |
| 12 | 2513 (56.5%) | 2309 (60.0%) | 842 (58.1%) | 0.252 |
| 13 | 2288 (51.4%) | 2102 (54.6%) | 784 (54.1%) | 0.245 |
| 15 | 2031 (45.7%) | 1839 (47.8%) | 674 (46.6%) | 0.504 |
| 17 | 1839 (41.3%) | 1718 (44.6%) | 601 (41.5%) | 0.131 |
|  |  |  |  |  |
| Body composition |  |  |  |  |
| 9 | 2832 (63.7%) | 2603 (67.6%) | 952 (65.7%) | 0.222 |
| 11 | 2587 (58.2%) | 2343 (60.9%) | 901 (62.2%) | 0.271 |
| 13 | 2246 (50.5%) | 2074 (53.9%) | 769 (53.1%) | 0.204 |
| 15 | 1950 (43.8%) | 1772 (46.0%) | 638 (44.1%) | 0.433 |
| 17 | 1768 (39.7%) | 1666 (43.3%) | 584 (40.3%) | 0.097 |
|  |  |  |  |  |
| Complete cases: height, weight, BMI, body composition | 986 (22.1%) | 940 (24.4%) | 325 (22.4%) | 0.140 |
| Complete cases: height, weight, BMI, body composition, confounders | 869 (19.6%) | 837 (21.8%) | 293 (20.2%) | 0.129 |
| Multiple imputed dataset with complete data on picky eating score | 4448 (100.0%) | 3848 (100.0%) | 1448 (100.0%) | - |
| Multiple imputed dataset with complete data on picky eating score, and at least one weight or height measurement, total n=7914 | 3529 (79.3%) | 3182 (82.7%) | 1203 (83.1%) | - |

**Supplementary Table 2**Details of multiple imputation dataset with complete data on picky eating score and at least one weight or height measurement, total n=7914

| **Variable** | **n imputed (%)** |
| --- | --- |
|  | Total n=7914 |
| Height |  |
| 7 | 1181 (15%) |
| 8 | 1894 (24%) |
| 9 | 1595 (20%) |
| 10 | 1696 (21%) |
| 11 | 1991 (25%) |
| 12 | 2250 (28%) |
| 13 | 2704 (34%) |
| 15 | 3370 (43%) |
| 17 | 3756 (47%) |
|  |  |
| Weight |  |
| 7 | 1191 (15%) |
| 8 | 2093 (26%) |
| 9 | 1540 (19%) |
| 10 | 1671 (21%) |
| 11 | 1990 (25%) |
| 12 | 2309 (29%) |
| 13 | 2748 (35%) |
| 15 | 3378 (43%) |
| 17 | 3756 (47%) |

**Supplementary Table 3**  Body composition from age 7 to 17 years for children identified as *very picky* eaters vs *not picky* eaters at 38 months: multiple imputation

| **Age (years)** | ***Not picky*** | ***Very picky*** | ***Not picky*** | ***Very picky*** | ***Not picky*** | | ***Very picky*** |  |
| --- | --- | --- | --- | --- | --- | --- | --- | --- |
|  | **Height (cm)** | | **Weight (kg)** | | **BMI (kg/m^2^)** | | | |
| **Boys** |  |  |  |  |  |  | | |
| 7 | 126.4 | 125.3 | 26.0 | 25.0 | 16.2 | | 15.9 |  |
| 8 | 133.3 | 132.0 | 30.6 | 29.6 | 17.1 | 16.7 | | |
| 9 | 140.2 | 139.0 | 35.0 | 33.5 | 17.7 | 17.2 | | |
| 10 | 144.4 | 143.2 | 38.3 | 36.8 | 18.3 | 17.8 | | |
| 11 | 150.7 | 149.6 | 43.6 | 42.0 | 19.1 | 18.7 | | |
| 12 | 157.3 | 156.6 | 49.0 | 47.8 | 19.7 | 19.3 | | |
| 13 | 164.6 | 164.1 | 55.0 | 53.8 | 20.2 | 19.8 | | |
| 15 | 172.8 | 172.1 | 63.9 | 62.6 | 21.3 | 21.1 | | |
| 17 | 176.7 | 175.9 | 71.7 | 69.8 | 22.9 | 22.5 | | |
|  |  |  |  |  |  |  | | |
| **Girls** |  |  |  |  |  |  | | |
| 7 | 125.5 | 124.3 | 26.1 | 24.7 | 16.5 | 15.9 | | |
| 8 | 132.4 | 130.9 | 30.8 | 29.1 | 17.4 | 16.9 | | |
| 9 | 139.7 | 138.2 | 35.6 | 33.4 | 18.1 | 17.4 | | |
| 10 | 144.6 | 142.8 | 39.4 | 36.9 | 18.7 | 17.9 | | |
| 11 | 151.9 | 150.2 | 45.5 | 42.6 | 19.6 | 18.7 | | |
| 12 | 157.9 | 156.7 | 50.8 | 55.4 | 20.3 | 19.5 | | |
| 13 | 162.7 | 161.9 | 55.4 | 53.3 | 20.9 | 20.3 | | |
| 15 | 166.1 | 165.6 | 60.4 | 58.3 | 21.9 | 21.3 | | |
| 17 | 166.7 | 166.1 | 64.5 | 61.7 | 23.2 | 22.4 | | |
|  | **Body fat (%)** | | **FMI (kg/m^2^)** | | **LMI (kg/m^2^)** | | | |
| **Boys** |  |  |  |  |  |  | | |
| 9 | 19.7 | 19.0 | 3.49 | 3.22 | 12.25 | 11.96 | | |
| 11 | 23.8 | 23.6 | 4.75 | 4.61 | 13.38 | 13.12 | | |
| 13 | 20.9 | 20.6 | 4.47 | 4.34 | 14.74 | 14.55 | | |
| 15 | 19.2 | 19.0 | 4.35 | 4.31 | 15.98 | 15.77 | | |
| 17 | 19.9 | 20.0 | 4.88 | 4.85 | 17.12 | 16.78 | | |
|  |  | |  | |  | | | |
| **Girls** |  |  |  |  |  |  | | |
| 9 | 25.5 | 24.3 | 4.56 | 4.19 | 11.39 | 11.23 | | |
| 11 | 28.1 | 26.9 | 5.67 | 5.20 | 12.89 | 12.59 | | |
| 13 | 27.7 | 26.9 | 6.01 | 5.64 | 13.72 | 13.52 | | |
| 15 | 29.3 | 28.5 | 6.69 | 6.28 | 14.08 | 13.85 | | |
| 17 | 32.2 | 31.1 | 7.82 | 7.24 | 14.17 | 13.85 | | |

Data shown for multiple imputation (ANOVA for difference between *not picky* and *very picky*).

Data presented as mean (95% CI and p values not provided in pooled output from SPSS).

**Supplementary Table 4** Models of effect of being a very picky eater at age 38 months on anthropometric variables from age 7 to 17 years old in the ALSPAC cohort (linear regression analysis): **multiple imputation**

| **Age (years)** | **Unstandardised B coefficient (95% CI)** | **P value** | **Unstandardised B coefficient (95% CI)** | **P value** | **Unstandardised B coefficient (95% CI)** | **P value** |
| --- | --- | --- | --- | --- | --- | --- |
|  |  |  |  |  |  |  |
|  | **Height (cm)** | | **Weight (kg)** | | **BMI (kg/m^2^)** | |
| **Boys** (n=2419) |  |  |  |  |  |  |
| 7 | -0.88 (-1.35, -0.40) | <0.001 | -0.58 (-0.91, -0.24) | 0.001 | -0.15 (-0.30, 0.012) | 0.071 |
| 8 | -1.05 (-1.52. -0.58) | <0.001 | -0.88 (-1.01, -0.45) | <0.001 | -0.24 (-0.42, -0.06) | 0.011 |
| 9 | -1.04 (-1.53, -0.54) | <0.001 | -0.99 (-1.55, -0.42) | 0.001 | -0.25 (-0.47, -0.03) | 0.027 |
| 10 | -0.98 (-1.50, -0.46) | <0.001 | -0.91 (-1.55, 0.26) | 0.006 | -0.21 (-0.45, 0.04) | 0.094 |
| 11 | -0.95 (-1.51, -0.39) | 0.001 | -0.91 (-1.69, -0.14) | 0.021 | -0.18 (-0.44, 0.09) | 0.196 |
| 12 | -0.67 (-1.37, 0.03) | 0.060 | -0.75 (-1.57, 0.07) | 0.074 | -0.14 (-0.41, 0.13) | 0.320 |
| 13 | -0.61 (-1.40, 0.18) | 0.129 | -0.73 (-1.60, 0.14) | 0.136 | -0.13 (-0.41, 0.15) | 0.355 |
| 15 | -0.73 (-1.48. 0.03) | 0.060 | -0.78 (-1.63, 0.67) | 0.071 | -0.09 (-0.36, 0.18) | 0.507 |
| 17 | -0.81 (-1.47, -0.15) | 0.016 | -1.33 (-2.26, -0.40) | 0.005 | -0.24 (-0.54, 0.06) | 0.114 |
|  |  |  |  |  |  |  |
| **Girls** (n=2313) |  |  |  |  |  |  |
| 7 | -1.09 (-1.59, -0.60) | <0.001 | -0.93 (-1.31, -0.55) | <0.001 | -0.31 (-0.49, -0.14) | 0.001 |
| 8 | -1.27 (-1.76, -0.78) | <0.001 | -1.11 (-1.58, -0.63) | <0.001 | -0.31 (-0.51, -0.11) | 0.002 |
| 9 | -1.32 (-1.86, -0.78) | <0.001 | -1.52 (-2.14, -0.91) | <0.001 | -0.44 (-0.68, -0.20) | <0.001 |
| 10 | -1.50 (-2.09, -0.91) | <0.001 | -1.55 (-2.29, -0.82) | <0.001 | -0.36 (-0.63, -0.10) | 0.008 |
| 11 | -1.54 (-2.16, -0.92) | <0.001 | -1.89 (-2.72, -1.05) | <0.001 | -0.43 (-0.71, -0.15) | 0.002 |
| 12 | -1.15 (-1.77, -0.52) | <0.001 | -1.69 (-2.54, -0.84) | <0.001 | -0.41 (-0.70, -0.12) | 0.006 |
| 13 | -0.76 (-1.37, -0.15) | 0.015 | -1.10 (-1.93, -0.26) | 0.010 | -0.23 (-0.52, 0.06) | 0.116 |
| 15 | -0.35 (-1.50, 0.36) | 0.335 | -1.13 (-1.97, -0.29) | 0.008 | -0.33 (-0.64, -0.02) | 0.040 |
| 17 | -0.54 (-1.15, 0.08) | 0.087 | -1.65 (-2.64, -0.65) | 0.001 | -0.45 (-0.80, -0.09) | 0.014 |
|  |  |  |  |  |  |  |
|  | **Body fat (%)** | | **FMI** | | **LMI** | |
| **Boys** (n=2419) |  |  |  |  |  |  |
| 9 | -0.31 (-1.18, 0.56) | 0.486 | -0.16 (-0.42, 0.10) | 0.234 | -0.27 (-0.68, 0.17) | 0.193 |
| 11 | 0.34 (-0.49, 1.16) | 0.427 | 0.02 (-0.20, 0.24) | 0.857 | -0.19 (-0.33, -0.05) | 0.007 |
| 13 | 0.19 (-0.66, 1.04) | 0.661 | 0.02 (-0.20, 0.24) | 0.855 | -0.14 (-0.34, 0.06) | 0.159 |
| 15 | 0.22 (-0.48, 0.92) | 0.534 | 0.09 (-0.14, 0.31) | 0.453 | -0.18 (-0.36, 0.00) | 0.054 |
| 17 | 0.44 (-0.32, 1.19) | 0.258 | 0.07 (-0.01, 0.03) | 0.165 | -0.28 (-0.43, -0.31) | <0.001 |
|  |  |  |  |  |  |  |
| **Girls** (n=2313) |  |  |  |  |  |  |
| 9 | -0.60 (-1.52, 0.31) | 0.198 | -0.20 (-0.48, 0.07) | 0.146 | -0.06 (-0.45, 0.33) | 0.756 |
| 11 | -0.58 (-1.38, 0.22) | 0.154 | -0.23 (-0.45, 0.00) | 0.051 | -0.19 (-0.33, -0.05) | 0.008 |
| 13 | -0.26 (-1.10, 0.58) | 0.544 | -0.12 (-0.37, 0.12) | 0.325 | -0.10 (-0.28, 0.07) | 0.245 |
| 15 | -0.45 (-1.17, 0.27) | 0.217 | -0.20 (-0.44, 0.05) | 0.117 | -0.13 (-0.28, 0.02) | 0.092 |
| 17 | -0.70 (-1.51, 0.10) | 0.087 | -0.35 (-0.64, -0.06) | 0.017 | -0.19 (-0.32, -0.06) | 0.006 |
|  |  |  |  |  |  |  |

Multiple regression analyses adjusted for maternal age, education, maternal BMI, birthweight, parity, breastfeeding, baseline BMI closest to 38 months, age at BMI measurement, age at each clinic

Reference category: *not picky*

R-M ANOVA in SPSS does not provide a pooled output for MI data.
